# Modularity and neuronal heterogeneity: Two properties that influence *in vitro* neuropharmacological experiments

**DOI:** 10.3389/fncel.2023.1147381

**Published:** 2023-03-20

**Authors:** Martina Brofiga, Fabio Poggio, Francesca Callegari, Mariateresa Tedesco, Paolo Massobrio

**Affiliations:** ^1^Department of Informatics, Bioengineering, Robotics, and Systems Engineering (DIBRIS), University of Genova, Genova, Italy; ^2^ScreenNeuroPharm S.r.l., Sanremo, Italy; ^3^3Brain GmbH, Wädenswil, Switzerland; ^4^National Institute for Nuclear Physics (INFN), Genova, Italy; ^5^MNESYS Extended Partnership Neuroscience and Neuropharmacology, Genova, Italy

**Keywords:** bicuculline, interconnected brain-regions-on-a-chip, cortical neurons, hippocampal neurons, dose-response curve, micro-electrode arrays (MEAs), neuromodulation

## Abstract

**Introduction:**

The goal of this work is to prove the relevance of the experimental model (*in vitro* neuronal networks in this study) when drug-delivery testing is performed.

**Methods:**

We used dissociated cortical and hippocampal neurons coupled to Micro-Electrode Arrays (MEAs) arranged in different configurations characterized by modularity (i.e., the presence of interconnected sub-networks) and heterogeneity (i.e., the co-existence of neurons coming from brain districts). We delivered increasing concentrations of bicuculline (BIC), a neuromodulator acting on the GABAergic system, and we extracted the IC_50_ values (i.e., the effective concentration yielding a reduction in the response by 50%) of the mean firing rate for each configuration.

**Results:**

We found significant lower values of the IC_50_ computed for modular cortical-hippocampal ensembles than isolated cortical or hippocampal ones.

**Discussion:**

Although tested with a specific neuromodulator, this work aims at proving the relevance of *ad hoc* experimental models to perform neuropharmacological experiments to avoid errors of overestimation/underestimation leading to biased information in the characterization of the effects of a drug on neuronal networks.

## 1. Introduction

Dissociated neuronal cultures coupled to Micro-Electrode Arrays (MEAs) are extensively used as *in vitro* model to study the mechanisms of the brain, such as the propagation of the signals (Callegari et al., 2022), to understand the progression of diseases, and to test the neuroactive and neurotoxic properties of different compounds delivered to neuronal tissues (Gramowski et al., 2000; Parviz and Gross, 2007). The first prototypes of MEAs developed by Gross (1979) were used as cell-based biosensors (Gross et al., 1992). In pioneering works, this *in vitro* model was used to quantify the effects of the delivery of neuromodulators (by means of perfusion systems or by pipetting them directly in the culture medium) on the spontaneous electrophysiological activity of neuronal networks. Martinoia et al. (2005) studied the effects of the delivery of agonists of ionotropic glutamate receptors belonging to the N-methyl-D-aspartate (NMDA) and α-amino-3-hydroxy-5-methyl-4-isoxazolepropionic (AMPA) to acute mature cortical networks by analyzing the drug-induced firing and bursting rate variations with respect to the spontaneous activity. Similarly, the effect of chronically delivered γ-aminobutyric acid (GABA), NMDA, and AMPA synaptic blockers at early stages of development was investigated to evaluate the state of hyperexcitability of cortical ensembles (Corner et al., 2005). Over the years, MEA-based systems have been proposed as testing platforms to explore the pharmacological and toxicological effects of numerous compounds on the spontaneous activity of excitable cells like neuronal and cardiac networks (Johnstone et al., 2010). It is worth mentioning how this approach dramatically reduces *in vivo* animal testing, resulting in susceptible, quick, and low cost neurotoxicological screening. Alloisio and colleagues evaluated the toxicological effects of pesticides on neuronal networks coupled to MEAs (Alloisio et al., 2015); 1 year later, the same research group exploited a similar experimental model to evaluate marine alga toxicity on mammalian cells (Alloisio et al., 2016). The success of these *in vitro* experimental models was boosted by the advancements of MEAs design. The state-of-the-art of MEA technology counts devices with up to thousands (Frey et al., 2009) or tens of microelectrodes organized in the form of multi-well chips that are available from different MEA suppliers (e.g., Multi-Channel Systems, Germany, 3Brain, Switzerland, Maxwell Biosystems, Switzerland, Axion Biosystems, US). The multi-well configuration allowed to simultaneously record the activity of independent populations treated with different compounds, or the same compound at different concentrations, reducing the time needed for data collection while increasing the statistical power of data analysis. In 2014, by using high-throughput 48-well MEA, a multi-laboratory study proved the reproducibility and the consistency of their experiments by using MEAs for neurotoxicological applications (Valdivia et al., 2014). Some years later, another multi-laboratory study demonstrated the power of neural networks coupled to MEAs as *in vitro* experimental model to test the effects of different compounds (Vassallo et al., 2017): the authors highlighted the robustness of the achieved results among the different involved laboratories despite significant methodological differences.

In parallel to pharmacological and toxicological applications, MEAs have been used to study the interactions of different interconnected neuronal populations. Kanagasabapathi et al. (2012) characterized the mutual interdependence between interconnected cortical and thalamic sub-populations, finding that the bursting activity of cortical assemblies is lower with thalamic inputs, and that the tonic spiking activity of the thalamic assembly displays shorter bursts thanks to the cortical afferences. Similarly, other researchers studied cortical-hippocampal circuits coupled to MEAs, where hippocampal neurons redistribute their connections when interconnected to cortical populations by projecting inhibitory links toward the cortical counterpart that modulate the temporal scale of the network bursts (Brofiga et al., 2022). These results suggest the possibility to observe different effects of a chemical compound in heterogeneous neuronal networks (with at least two different neuronal types) with respect to isolated ensembles (the state of the art of neuropharmacological experiments). Drug testing performed on homogeneous neuronal cultures could produce results far from those of real *in vivo* condition; therefore, the effects of the drug itself could be underestimated or overestimated, leading to distorted if not incorrect information (Brofiga and Massobrio, 2022).

Our main goal was to prove that the response of an *in vitro* neuronal model to the delivery of a chemical compound changes as a function of its degree of complexity. More specifically, we tested our hypothesis gradually integrating different aspects of the *in vivo* microenvironment, like modularity and heterogeneity, and proved that they cause different neuropharmacological-induced outcomes.

In particular, we exploited a two-compartment polydimethylsiloxane (PDMS) mask coupled to MEAs to recreate modular networks (Sporns and Betzel, 2016) made up of interconnected cortical and hippocampal sub-populations. The aim was to assess the influence of the model itself on the evaluation of the drug’s effect by comparing drug-induced variations (as a function of the concentration) in the electrophysiological activity of heterogeneous cultures with those observed in homogeneous controls. We tested such hypothesis by considering bicuculline (BIC), a competitive antagonist of GABA_A_ receptors (Razet et al., 2000). The main achieved result is a statistically significant difference in the IC_50_ values (i.e., the effective concentration yielding a reduction in the response by 50%) of the mean firing rate (MFR) when computed in interconnected cortical-hippocampal ensembles compared to isolated cortical or hippocampal ones, supported by a different remodulation of the detected inhibitory functional connections.

## 2. Materials and methods

### 2.1. Polydimethylsiloxane mask

**FIGURE 1:**
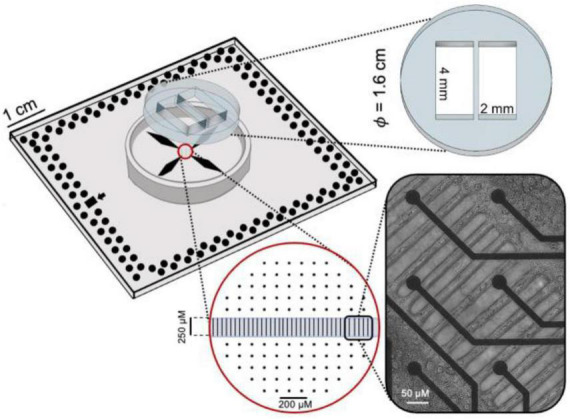
Sketch of the PDMS mask coupled to MEA where the geometrical features are reported. On the bottom right corner, a differential interference contrast image of the neurites passing through the microchannels.

The polydimethylsiloxane (PDMS) mask consisted of two compartments 4 mm wide and 2 mm long (Figure 1). The compartments were connected by means of a 56 microchannel array, regularly spaced (50 μ*m*). The single microchannel was 10 μ*m* in width, 250 μ*m* in length and 5 μ*m* in height. The size of the microchannels prevented the migration of cells between the compartments while allowing the passage of neurites only (Taylor et al., 2003). The mask was fabricated by conventional soft lithography molding techniques. A mixture of PDMS prepolymer and curing agent (10:1 w/w), was allowed to polymerize for 10 min at 80°C. Masks were aligned and reversibly bounded to planar MEAs with 120 electrodes, 30 μ*m* in diameter and 200 μ*m* in spacing, arranged in a 12 × 12 array. The assembled device (i.e., MEA with PDMS mask) was sterilized in a dry oven at 120°C for 3 h. Finally, oxygen-plasma treatment (60 s at 120 W) was performed to hydrophilize the microchannels selectively.

### 2.2. Cell culture preparation

All the procedures executed for the preparation of cell cultures for experimental purposes were carried out in accordance with the European Animal Care Legislation (2010/63/EU), and in compliance with the legislative decree of the Ministry of Health (DL 116/1992) and the guidelines of the University of Genova (Prot. 75F11.N.6JI, 08/08/18), in order to reduce the number of animals needed for testing and their suffering. We used cultures of dissociated cortical and hippocampal neurons of embryonic rats, at gestational day 18−19 (E18-E19). The cerebral cortices/hippocampi of embryos were dissected and underwent a first stage of dissociation by enzymatic digestion solution with Trypsin (Sigma-Aldrich, USA) at 0.125% and DNAse (Sigma-Aldrich, USA) 0.02 mg/ml, diluted in Hank’s solution (GIBCO Invitrogen), for 20 min at 37°C. The digestion was stopped by adding medium (Neurobasal, Gibco) complemented with 10% FBS (fetal bovine serum, Sigma-Aldrich, USA). The enzymatic dissociation was followed by mechanical trituration with a fine-tipped pipette. The resulting cellular suspension was diluted in Neurobasal medium supplemented with 2% B-27 Supplement (GIBCO Invitrogen), 1% stable L-Glutamine (GlutaMAX 100×, GIBCO Invitrogen) and 1% PenStrep (Penicillin-Streptomycin Solution, GIBCO Invitrogen). No antimitotic drug was added to prevent glia proliferation, since glia cells are known to be fundamental for the healthy development of neuronal populations (Pfrieger and Barres, 1997). Finally, cells were plated directly inside the two interconnected compartments coupled to 120-channel MEAs (Multi Channel Systems, Germany, MCS) previously sterilized and coated with poly-L-ornithine (100 μg/ml, Sigma-Aldrich, USA). Cortical and hippocampal neurons were plated at the density of 1,500 cells/mm^2^ and 1,300 cells/mm^2^, respectively. The biological samples were incubated at 37°C, 5% CO_2_, 95% humidity. For the main dataset, half volume of the medium was replaced with BrainPhys medium (StemCell Technologies) supplemented with 2% NeuroCult SM1 (StemCell Technologies), 1% Glutamax and 1% PenStrep solution 5 days after the plating. Half of the medium was changed weekly allowing the neurons to organize into a morphologically and functionally mature network within 3 weeks.

### 2.3. Drug delivery protocol

To evaluate the effects of the bicuculline (BIC, Sigma-Aldrich, USA) on the spontaneous network activity, the drug was tested with increasing concentrations by directly pipetting it into the culture medium. A wide administration scale (100 nM–500 μM) with significant points in the logarithmic scale was chosen to quantify the effects on the neuronal activity. For each concentration, the electrophysiological activity was recorded (sampling frequency of 10 kHz) for 10 min.

### 2.4. Dataset

The effects of BIC on the electrophysiological activity were evaluated over a dataset of *n* = 6 modular cortical-hippocampal (CxHp) cultures. Four configurations of control experiments were performed to dissect the contributions of modularity and heterogeneity. In particular, we delivered the same BIC concentrations to homogeneous cortical (Cx, *n* = 6) and hippocampal (Hp, *n* = 7) populations without modularity, and to modular homogeneous cortical (CxCx, *n* = 5) and hippocampal (HpHp, *n* = 5) populations. The *n* = 29 networks came from three preparations, each exploited to plate both heterogeneous and homogeneous cultures. For each network, the basal activity (i.e., without BIC) was recorded for 10 min, and used as reference to normalize all the other phases, therefore allowing the comparison between different cultures. The increasing concentrations of BIC were sequentially applied to the culture by directly pipetting the drug solution in the medium, and the network activity was recorded for 10 min at each concentration. To avoid observing mechanical effects due to the administration of the compound or seeing the transient effect due to diffusion processes, the first 2 min of each phase were discarded. Therefore, the analyses were performed on 8 min recordings for each phase (i.e., BIC concentration). All the recordings were performed after 18 days *in vitro* (DIVs), which corresponds to a mature stage of development of the networks (Bardy et al., 2015). The electrophysiological activity was acquired using the MEA2100 system (MCS). After a period of acclimatation of about 10 min, the spontaneous activity was recorded in stable conditions: 37°C, and 5% CO_2_.

### 2.5. Data analysis

Raw data were spike-detected using the Precise Timing Spike Detection algorithm (Maccione et al., 2009). The algorithm required the setting of three parameters: (I) a differential threshold value, which is set independently for each channel and calculated as eight times the standard deviation of the signal’s biological and thermal noise; (II) a peak lifetime period, related to the duration of a spike (set at 2 ms); (III) a refractory period, associated to the minimum interval between two consecutive events (set at 1 ms). Raw data were not spike sorted, since spike sorting did not significantly increase the spatial reconstruction of the network when we sampled the activity of a few thousand neurons. A channel was considered active if it recorded at least one spike in 10 s (MFR > 0.1 sp/s). During the drug delivery protocol, the MFR value of each culture was averaged on the number of active electrodes during the basal recording (initial conditions). Then, the MFR values of each experiment were normalized with respect to the corresponding values of the reference (basal) activity: such a procedure allowed to compare the MFR values of the different experiments. The variation of the normalized MFR as a function of the concentration was fitted by the Hill equation (Eq. 1), a widely-used model to analyze nonlinear drug concentration–response relationships (Wagner, 1968).


(1) $$M\,F\,R_{n\,o\,r\,m}\,\left(\left[B\,I\,C\right]\right)=M\,F\,R_{n\,o\,r\,m}^{m\,a\,x}+\frac{M\,F\,R_{n\,o\,r\,m}^{m\,i\,n}-M\,F\,R_{n\,o\,r\,m}^{m\,a\,x}}{1+10^{H\,C\,\left(\log\left(I\,C_{50}\right)-\log\left(\left[B\,I\,C\right]\right)\right)}}$$

In Eq. (1), $M\,F\,R_{n\,o\,r\,m}^{m\,i\,n}$ and $M\,F\,R_{n\,o\,r\,m}^{m\,a\,x}$ are the highest and the lowest normalized MFR values, respectively. *HC* is the Hill coefficient which provides the largest absolute value of the slope of the curve. Finally, IC_50_ is the effective concentration yielding a reduction in the response by 50%.

In order to appreciate the variations induced by the BIC on network connectivity, the Total Spiking Probability Edge (TSPE) algorithm was employed (De Blasi et al., 2019) to identify the functional connections and to discriminate the excitatory from the inhibitory ones. Once computed the connectivity matrix, we discarded the values that did not have a physiological significance in terms of propagation speed. Therefore, we applied a spatial filter to maintain functional connections with a propagation speed between 30 and 400 mm/s (Jacobi and Moses, 2007). Then, we set up an independent hard threshold for excitatory and inhibitory connections (Poli et al., 2016):


(2) $$T\,H_{e,i}=\mu_{e,i}\pm n_{e,i}\,\cdot \,\sigma_{e,i}$$

where μ and σ are the mean and the standard deviation of all the non-zero elements of the cross-correlation matrix, and *n* is an integer. The subscripts *e* and *i* are relative to the excitatory and inhibitory links, respectively. We chose *n* in order to keep an average percentage of inhibitory links of about 20−30% in the basal phase, consistent with physiological values (Marom and Shahaf, 2002). The results presented in Section “3.3. Bicuculline modulates differently inhibitory functional connections in heterogeneous and homogeneous networks” consider the functional connections detected during the basal condition as reference. Thus, we quantified the variation of the percentage of the initial inhibitory links as a function of the BIC concentrations.

All the algorithms were developed in Matlab (The Mathworks, Natick, MA, US) and Python (Python Software Foundation, Wilmington, DE, US). Statistical analysis was performed using Python. Since data do not follow a normal distribution (evaluated by the Kolmogorov–Smirnov normality test), we performed a non-parametric Kruskal–Wallis test. Significance levels were set at *p* < 0.05. The box plots representation indicates the percentile 25−75 (box), the standard deviation (whiskers), the mean (square), and the median (line) values.

## 3. Results

We explored the effect of increasing concentrations of BIC on modular cortical and hippocampal ensembles (CxHp) compared to homogeneous cortical and hippocampal (with and without modularity) controls.

### 3.1. BIC modulates the dynamics of cortical-hippocampal assemblies

The spontaneous electrophysiological activity of *in vitro* CxHp networks showed a wide repertoire of activity patterns characterized by the simultaneous presence of spiking and bursting events, which may involve the entire network in the form of network bursts (Brofiga et al., 2022). The release of a neuromodulator like BIC induced a change in the balance between the excitatory and inhibitory components of the network (Razet et al., 2000), which caused a less organized, more scattered spiking activity, and a redistribution of the bursting dynamics.

**FIGURE 2:**
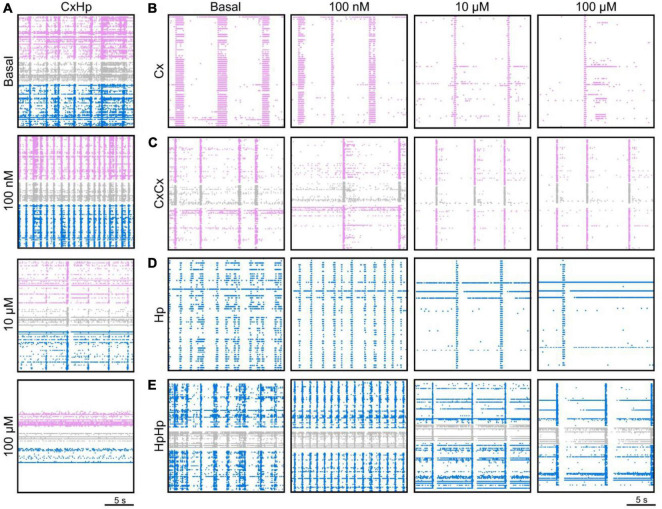
The delivery of different concentrations of bicuculline (BIC) modulates the firing activity of both homogeneous and heterogeneous neuronal assemblies. A total of 20 s electrophysiological activity recorded at DIV 18 of a representative **(A)** cortical-hippocampal assembly during its spontaneous activity (basal) and when 100 nM, 10 μM, and 100 μM of BIC were delivered; **(B)** homogeneous and **(C)** modular homogeneous cortical control, **(D)** homogeneous and **(E)** modular homogeneous hippocampal control. Pink and blue dots identify the detected spikes of the cortical and hippocampal populations, respectively, while gray dots identify the activity generated by the neurites passing through the microchannels.

**FIGURE 3:**
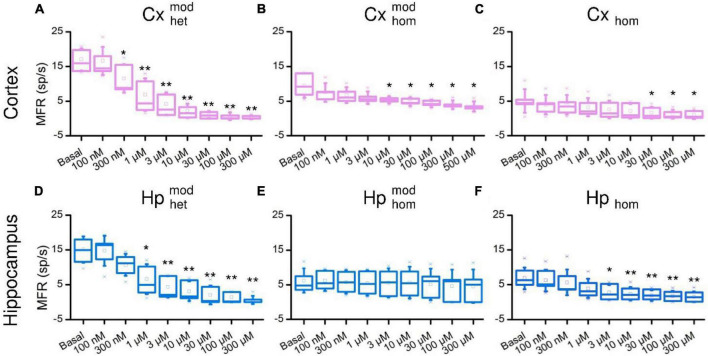
Firing rate distributions of the cortical (top) and hippocampal (bottom) ensembles in the different experimental configurations as a function of the increasing BIC concentration. Cortical cells in **(A)** heterogeneous, **(B)** homogeneous and modular, **(C)** homogeneous configurations. Hippocampal cells in **(D)** heterogeneous, **(E)** homogeneous and modular, **(F)** homogeneous configurations (* refers to 0.01 < *p* < 0.05, **0.001 < *p* < 0.01, Kruskal–Wallis, nonparametric test).

The raster plots of Figure 2A are relative to a representative CxHp assembly during its initial spontaneous activity (basal, *top panel*), and after the delivery of 100 nM, 10 μM, and 100 μM BIC solution. From a qualitative point of view, the increased doses of BIC induced a change in the pattern of activity: the spiking distribution of both cortical (pink) and hippocampal (blue) sub-populations was increasingly scattered with higher concentrations of BIC. The same behavior was observed in both non-modular (Figures 2B-D) and modular (Figures 2C-E) controls. However, BIC had a significant effect on the firing rate of the network already at low concentrations in the case of heterogeneous populations: the cortical sub-population influenced by hippocampal input (${\text{Cx}}_{\text{het}}^{\text{mod}}$) showed a faster MFR decrease as a function of the BIC concentration than in the controls. In ${\text{Cx}}_{\mathrm{het},}^{\text{mod}}$ the first significant reduction (−59.6% compared to the basal phase, *p* = 0.04) was observed at the concentration of 300 nM (Figure 3A). In the case of homogenous controls, with (${\text{Cx}}_{\text{hom}}^{\text{mod}}$) and without (Cx_hom_) modularity, the progressive reduction of the firing rate was observed too, but the first significant difference with respect to the basal condition was observed at higher concentrations: the MFR of the ${\text{Cx}}_{\text{hom}}^{\text{mod}}$ decreased by −43.7% (*p* = 0.03) at 10 μM (Figure 3B), while in Cx_hom_ it showed a reduction of −66.4% (*p* = 0.04) at 30 μM (Figure 3C). If on one hand the cortical subpopulation had a similar trend among the different configurations, albeit the marked shift in the necessary dosage to induce a modulation of the activity, on the other, the hippocampal counterpart behaved in a slightly different way. Hippocampal neurons in the heterogeneous configuration (${\text{Hp}}_{\text{het}}^{\text{mod}}$) showed a quick reduction in the MFR, with a behavior similar to the interconnected cortical sub-population (Figure 3A). The first statistical change in the firing rate with respect to the basal phase occurred at 1 μM (Figure 3D), with a reduction of −50.7% (*p* = 0.02). Such a behavior also occurred in the non-modular homogeneous control (Hp_hom_), albeit at a higher concentration: −58.6% (*p* = 0.02) at 3 μM (Figure 3F). This outcome highlighted an earlier onset of the effects of the BIC in the CxHp configuration also in the hippocampal sub-population. A different behavior was observed in the modular homogeneous hippocampal assembly (${\text{Hp}}_{\text{hom}}^{\text{mod}}$), where the firing rate showed no statistical variations regardless of BIC concentration (Figure 3E).

### 3.2. Comparison between dose-response curves of heterogeneous and homogeneous networks

To quantify the effects of BIC on the electrophysiological activity, we computed the resulting MFR dose-response curves for the different experimental configurations, and from their fitting (Eq. 1), we extracted the corresponding IC_50_ values.

**FIGURE 4:**
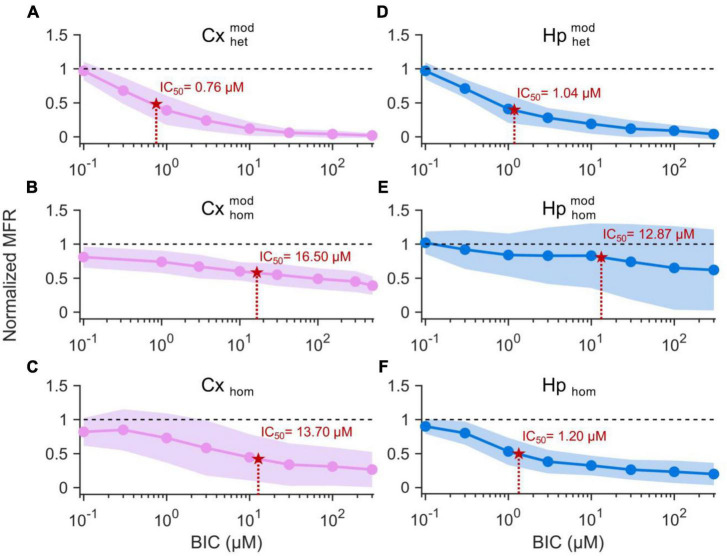
Dose-response curves of the different experimental configurations. On the left, cortical sub-populations in the **(A)** heterogeneous, **(B)** modular homogeneous, and **(C)** homogeneous configurations. On the right, hippocampal sub-populations in the **(D)** heterogeneous, **(E)** modular homogeneous, and **(F)** homogeneous configurations. The solid lines represent the average Hill fitting curves (Eq. 1), while the shadow the standard deviation over the performed experiments. IC_50_ values are depicted with red stars and are superimposed to the curves.

A decreasing sigmoidal fitting function characterized the dose-response curves of the cortical sub-population when interconnected to the hippocampal one (CxHp configuration) with a coefficient of determination (*R*^2^) equal to 0.98 ± 0.02 (Figure 4A). A similar behavior was found in both the homogeneous CxCx (Figure 4B) and Cx (Figure 4C) configurations, with a coefficient of determination equal to 0.84 ± 0.09 and 0.98 ± 0.02, respectively. We observed a slower reduction in the firing activity in the two controls than in the heterogeneous configuration with the increase of the BIC dosage. Indeed, in ${\text{Cx}}_{\text{hom}}^{\text{mod}}$ and Cx_hom_, a normalized MFR value of less than 0.5 was detected at higher concentrations, underlining how heterogeneity anticipated the effects of the drug. Furthermore, the absence of modularity in the cortical population induced a greater variability in the obtained dose-response curves: the standard deviation of the normalized MFR ranged between 0.03 and 0.21 in the CxHp configuration, 0.13 and 0.17 in the CxCx, and 0.20 and 0.40 in the Cx configuration.

Also the hippocampal sub-population responded to the delivery of increasing concentrations of BIC with a decreasing sigmoidal Hill function both in the CxHp (*R*^2^ = 0.95 ± 0.05) and in the Hp (*R*^2^ = 0.97 ± 0.02) configurations (Figures 4D-F). The sigmoidal response was weaker in the HpHp, as supported by the low value of *R*^2^ (0.74 ± 0.38, Figure 4E). In the hippocampal assembly, modularity alone induced a delayed response to the drug (Figure 4E) compared to the non-modular Hp configuration. Indeed, even by considerably increasing the BIC dosage, the firing activity trend was almost flat, and no strong variations were observed. Differently from what observed in the cortical assemblies (Figure 4B), the modularity alone induced an increase in the variability of the experiment results in the hippocampal sub-population (Figure 4E): the standard deviation laid between 0.16 and 0.21 in the HpHp configuration, and between 0.10 and 0.20 in the non-modular one. Such effects disappeared with the introduction of the cortical sub-population (heterogeneity), where the influence of BIC was already visible at low concentrations (about 1 μM) and where it induced a reduction in the interval of the standard deviation (between 0.07 and 0.20).

**FIGURE 5:**
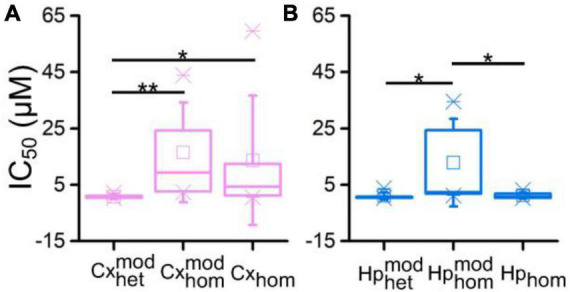
IC_50_ distributions of **(A)** cortical and **(B)** hippocampal networks as a function of the different experimental configurations (* refers to 0.01 < *p* < 0.05, **0.001 < *p* < 0.01, Kruskal–Wallis, nonparametric test).

In order to quantify the previous observations, we extracted the numerical values of the IC_50_ of the different configurations, and we evaluated (possible) differences between heterogeneous and homogeneous cultures with and without modularity. The cortical sub-population in the CxHp configuration showed a IC_50_ value of 0.76 ± 0.75 μM, statistically different from the values observed in the absence of hippocampal inputs. In particular, in the case of modular homogeneous cultures, the IC_50_ value was equal to 16.50 ± 17.74 μM, a value greater than in the CxHp configuration by +2,071% (*p* = 0.006, Figure 5A). Also in the non-modular control (Cx) the IC_50_ (13.70 ± 22.95 μM) was higher than in the CxHp cultures by +1,703% (*p* = 0.02, Figure 5A). While the IC_50_ values of the cortical sub-population seemed to be mostly influenced by the presence of the hippocampus, the behavior of the hippocampal sub-population was characterized by more complex relationships. In the heterogeneous configuration, the detected IC_50_ value of the hippocampal subpopulation was equal to 1.04 ± 1.32 μM. The lack of cortical input (HpHp) induced a shift forward of the effects of BIC (12.87 ± 15.56 μM), which determined a positive variation of +1,138% (*p* = 0.03) compared to the CxHp configuration. On the other hand, in the homogeneous controls the absence of modularity induced a reduction of −90% (1.20 ± 1.04 μM) in the value of IC_50_ (*p* = 0.04, Figure 5B), compared to HpHp, anticipating the BIC modulation in the hippocampal assembly.

Finally, for each configuration, we estimated the reproducibility of the BIC effect on the IC_50_ by evaluating the Fano Factor (FF, computed as the variance of the IC_50_ values over the mean value, Table 1). Cortical sub-population in the CxCx and Cx controls showed FFs greater than in the CxHp configuration by 2,629% and 5,400%, respectively. On the other hand, the hippocampal sub-population in two different configurations was characterized by low FFs: we observed a FF equal to 1.7 μM in the heterogeneous networks and equal to 0.7 μM in the non-modular homogeneous ones. A different situation was observed in the modular homogeneous configurations characterized by a FF 1,006% greater than the FF of the ${\text{Hp}}_{\text{het}}^{\text{mod}}$.

**TABLE 1:** Fano factor (FF) values of IC_50_ in the different configurations.

Sub-population	Heterogeneous configuration	Modular homogeneous	Non-modular homogeneous
Cortical	0.7 μM	19.1 μM	38.5 μM
Hippocampal	1.7 μM	18.8 μM	0.9 μM

### 3.3. Bicuculline modulates differently inhibitory functional connections in heterogeneous and homogeneous networks

In this Section, we investigated whether the different behavior exhibited by the CxHp and control networks upon delivering the BIC (Figures 4, 5) might have been induced by a different reorganization of the inhibitory functional connections. In this perspective, we analyzed the variation of the delicate balance between the excitatory and inhibitory components of the network by means of the TSPE algorithm (Section “2.5. Data analysis”).

**FIGURE 6:**
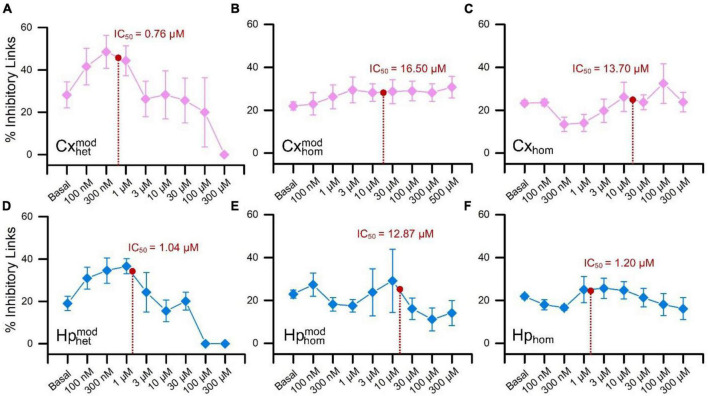
Percentage of the detected functional inhibitory links as a function of the increasing concentrations of BIC. The top row is relative to the cortical populations, while the bottom to the hippocampal one. Cortical sub-populations in **(A)** heterogeneous, **(B)** modular homogeneous, and **(C)** homogeneous configurations. Hippocampal sub-populations in **(D)** heterogeneous, **(E)** modular homogeneous, and **(F)** homogeneous configurations. For the sake of readability, the IC_50_ values were superimposed on each curve (red dot).

In the case of heterogeneous CxHp cultures, we observed two distinct phases: the first one characterized by a growth of the percentage of inhibitory links (at low concentrations of BIC, i.e., up to 300 nM), and the second one by a slow decrease (Figures 6A, D). In particular, in the cortical sub-population, the decreasing phase occurred between 300 nM and 1 μM, i.e., in the range of concentrations where the value of IC_50_ was identified (0.76 ± 0.75 μM). Differently, in the hippocampal sub-population the beginning of the reduction in the percentage of inhibitory links was shifted forward, between 1 μM and 3 μM, a range which contains the hippocampal IC_50_ value (1.04 ± 1.32 μM). Therefore, the two interconnected populations display a similar trend, although shifted as a function of the detected IC_50_ values. On the contrary, the behavior observed in both modular and non-modular homogeneous controls was different. In both homogeneous configurations, the cortical population did not show any evident increasing/decreasing trend (Figures 6B, C), reflecting the behavior observed in the firing values of the dose-response curves (Figures 4B, C). Furthermore, in the modular case (Figure 6B), the percentage of inhibitory links showed weak variations (less than 10%), providing no information on the possible functional effects of the BIC. Also in the homogeneous hippocampal sub-population the observed behavior was inconclusive (Figures 6E, F). In particular, in the modular controls (Figure 6E) an irregular trend of the percentage of inhibitory connections emerged, probably induced by the significant variability of the firing (Figure 4E). On the other hand, in the non-modular configuration, minimal variations were observed (less than 5%), not allowing to infer any information on the effect of BIC.

### 3.4. Medium culture and degree of maturation influence the effect of BIC

Differently from other works (Thompson, 1993; Forti et al., 1997; Bi et al., 2013) where BIC induced an increase in the excitability of neuronal networks with a boost in the firing rate (Morefield et al., 2000; Colombi et al., 2013), in the present work, such an effect was not observed in any of the experimental conditions. On the contrary, our experiments showed a clear decrease in the MFR as a function of the concentration of delivered BIC (Figures 3, 4).

In this Section, we showed that this opposite effect could depend on the level of maturation of the network. To test this hypothesis, we performed two additional experiments. Considering that it was proven that BrainPhys with SM1 supplement (StemCell Technologies) speeds up neuronal development and determines a higher electrophysiological activity rate than Neurobasal with B27 supplement (Gibco), the golden standard neuronal medium (Bardy et al., 2015), we repeated the drug delivery protocol (cf., Section “2.3. Drug delivery protocol”) on networks matured in Neurobasal/B27 at the same DIV (Cx_Ne_ and Hp_Ne_). Similarly, we performed the same protocol on networks cultured in BrainPhys/SM1 (Cx_*Br*_, Hp_Br_) but on an early stage of maturation, i.e., at DIV 11 (Cx_Br_ and Hp_Br_).

**FIGURE 7:**
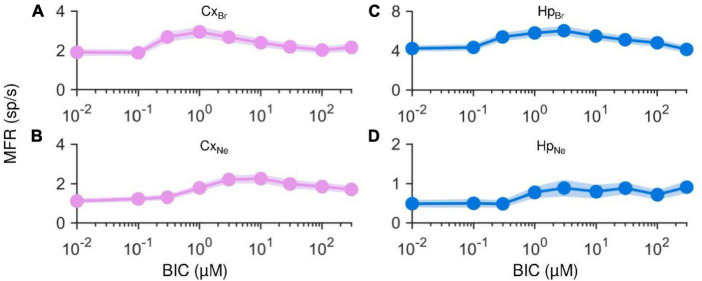
Mean and standard error of the mean of single electrode MFRs computed throughout the entire drug delivery protocol for each experimental condition. On the left, cortical networks matured in **(A)** BrainPhys/SM1 until DIV 11, and **(B)** Neurobasal/B27 until DIV 18. On the right, hippocampal networks matured in **(C)** BrainPhys/SM1 until DIV 11, and **(D)** Neurobasal/B27. Each averaged value is represented by a circle and is given by the mean of all single electrode MFRs of the same experimental condition corresponding to the same BIC concentration.

First, it is worth noticing how the average starting firing values in both cortical (Figure 7A) and hippocampal (Figure 7C) cultured in BrainPhys/SM1 but recorded at DIV11, and in both cortical (Figure 7B) and hippocampal (Figure 7D) networks grown on Neurobasal/B27 medium were markedly lower than those observed in the homogenous cortical and hippocampal cultures matured in BrainPhys/SM1 at DIV 18 (Figures 3A-D). In particular, the Cx_Br_ assemblies (Figure 7A) showed a baseline reduction of −62.1% and Cx_Ne_ of −77.8% (Figure 7B). Also in the hippocampal networks a reduction of −38.4 and −92.8% could be observed in Hp_Br_ (Figure 7C) and in Hp_Ne_ (Figure 7D), respectively. Analyzing the effects of the BIC in the modulation of the electrophysiological activity, a global increase in the MFR values in all the considered configurations was observed, with some differences at higher concentrations. Regardless of the medium, with a young network (Neurobasal/B27 at DIV 18 or BrainPhys/SM1 at DIV 11), cortical assemblies showed a global increase in activity rate, reaching a maximum value at 1 μM and 10 μM, respectively. Subsequently, as the concentration of BIC increased, the activity remained unchanged, reaching a sort of plateau. The behavior of the hippocampal network was different: in BrainPhys/SM1 at DIV 11, an increasing phase was followed by a decreasing one, with a peak at 3 μM and a subsequent mild decrease (Figure 7C). On the other hand, the Hp_Ne_ at DIV 18 showed a gradual and continuous increase of the firing rate, reaching its maximum value at 300 μM. The key point of these analyses was that both cortical and hippocampal networks matured in BrainPhys/SM1 until DIV 11 or in Neurobasal/B27 until DIV 18 showed the canonical firing increase due to the BIC effects.

## 4. Discussion and conclusion

In the present work, we aimed at opening a new scientific question in the field of experimental neuroscience and neuropharmacology. By exploiting the theoretical knowledge gained over the years and the neurotechnologies available nowadays, are the widely used *in vitro* network experimental models (i.e., large-scale homogeneous neuronal networks) the best choice to investigate the effects of a neuropharmacological manipulation? In this work, we performed experiments to highlight how *in vitro* neuronal models with different degrees of complexity (only modularity, modularity and heterogeneity) and a different degree of maturation (i.e., development) could induce different responses to the delivery of a well-known neuromodulator like the bicuculline (BIC). We tested the effect of increasing doses of BIC, a competitive antagonist of GABA_A_ receptors (Razet et al., 2000) in heterogeneous neuronal assemblies made up of interconnected cortical and hippocampal neurons whose modular connectivity (Sporns and Betzel, 2016) was engineered by means of polymeric structures (Figure 1). The quantification of the effects was derived by means of dose-response curves, which allow mathematically computing the IC_50_ parameter that indicates the half maximal inhibitory concentration.

### 4.1. Neuronal heterogeneity shifts the effect of BIC

Our results showed a clear and statistical difference in IC_50_ values when BIC was delivered to cortical-hippocampal or to homogeneous controls (i.e., made up of only one neuronal type), which is the typical experimental model (Figure 4). When interconnected to hippocampal ensembles, cortical neurons showed an average IC_50_ of 0.76 ± 0.75 μM, statistically different from the values observed in the absence of hippocampal inputs or when cortical neurons are not arranged in a modular topology (Figure 5A). This evidence suggests how both the connectivity of the network (here engineered by means of a polymeric mask) and its neuronal composition (two neuronal types in the present work) shape its response as indicated by the significant shift of the IC_50_ values. The behavior of the hippocampal ensembles is similar, although in this case no significant differences were detected between the heterogeneous and non-modular homogeneous configurations (Figure 5B). Also, the analysis of the functional connectivity underlines the central role of the heterogeneity in an *in vitro* neuronal network model. A marked effect of BIC on the distribution of inhibitory links was found only in CxHp networks in correspondence of IC_50_ values (Figures 6A, D). Instead, in the homogeneous configurations, it is practically negligible, confirming the trends of the dose-response curves, which display a moderate sigmoidal trend, (Figures 3B, F) characterized either by a low goodness-of-fit or with a high experimental variability (Figures 3C, E) and high Fano Factor values (Table 1).

### 4.2. The degree of maturation of complex ensembles changes the BIC effect

Differently from other works performed both *in vivo* (Dinocourt et al., 2011) and *in vitro* (Thompson, 1993; Forti et al., 1997; Bi et al., 2013), in the present work the boosting effect of the BIC was not observed in any of the experimental conditions. On the contrary, our experiments showed a clear decrease in the MFR as a function of the concentration of delivered BIC (Figures 3, 4). This opposite effect could depend on the degree of maturation of the network that is also influenced by the type of culture medium where neurons grow. We investigated this possibility by performing control experiments using cortical and hippocampal networks matured in BrainPhys/SM1 compared to one developed in Neurobasal/B27 at the same DIV (cf., Section “3.4. Medium culture and degree of maturation influence the effect of BIC”). We found that networks matured in Neurobasal/B27 showed an increase in the electrophysiological activity due to the BIC delivery as reported in the literature. These results could be explained as in the following: (i) the neuronal medium is a fundamental factor that determines a variability in the global gene expression in cells (Guo et al., 2016), which means that different neuronal culture media (BrainPhys/SM1 vs. Neurobasal/B27, (Jackson et al., 2018)) might differently affect protein expression in neurons, thus altering the neuronal maturation of the network; (ii) consequently, the different degree of network development determines a different degree of spontaneous electrophysiological activity (Wagenaar et al., 2006). Our results showed that both Cx and Hp networks matured in BrainPhys until DIV 11 (Figures 7A, B) and in Neurobasal recorded at DIV 18 (Figures 7C, D) displayed the canonical increase in the firing rate as effect of BIC administration, suggesting that these networks might have reached the same degree of maturation. Although such effect varied depending on the neuronal type of the cultures, it is evident that in all the networks matured in BrainPhys and recorded at DIV 18 the firing rate decreased with the increase of the concentration of BIC (Figure 4). Finally, it is worth noticing that Cx_Ne_, and Hp_Ne_ spontaneous firing rate and Cx_Br_ and Hp_Br_ recorded at DIV 11 (Basal condition, Figure 7), was always lower than 5 sp/s, contrary to the other cultures matured in BrainPhys/SM1 until DIV 18 where basal MFR was greater than 10 sp/s.

### 4.3. Exploitation of complex brain-on-a-chip models

The present work proved the relevance of an accurate biological neuronal model when neuropharmacological experiments are performed. The conventional experimental protocols for this kind of investigations make use of simple homogeneous neuronal networks with only cortical or hippocampal neurons randomly grown over MEAs. However, such oversimplifications (i.e., the lack of a modular connectivity or heterogeneity in the network composition) can lead to under- or over-estimate the response to the drug, leading to biased information (Bang et al., 2019). The considered case-study (i.e., delivery of BIC) is a proof of the possible alterations in the evaluation of the effects of a drug by simply adding two neuronal populations with a modular connectivity. In addition, we observed the relevance of when such a chemical manipulation is performed. Depending on the stage of development, cortical and hippocampal assemblies can display opposite effects (Figure 7). In light of the achieved results, we do not have the claim to assert that the proposed model is the best one, our take-home message is the need for a more in-depth and precise study on the kind of model to use when testing drug effects on neuronal tissues. It is worth mentioning that the human brain is made up of about 86 billion neurons that differ in structure and function (heterogeneity) and are organized following precise connectivity rules (modularity) in a 3D fashion (not taken into account in this work). These three key ingredients identify a new engineered system, in which neurons can live, grow, and connect to establish intricate connectivity, which can be coupled to integrated micro-transducers and that can be exploited to perform realistic and not oversimplified experiments (Brofiga et al., 2021).

## Data availability statement

The data presented in this study are deposited in the Zenodo repository, accession number: https://doi.org/10.5281/zenodo.7665283.

## Ethics statement

All the procedures executed for the preparation of cell cultures for experimental purposes were carried out in accordance with the European Animal Care Legislation (2010/63/EU), and in compliance with the legislative decree of the Ministry of Health (DL 116/1992) and the guidelines of the University of Genova (Prot. 75F11.N.6JI, 08/08/18), in order to reduce the number of animals needed for testing and their suffering.

## Author contributions

MB, FP, and FC performed the experiments. FP developed the software tool for extracting the dose-response curves. MB performed the analysis and prepared the figures of the manuscript. MT performed the rat dissections and provided the dissected cells. PM conceived and supervised the work. All authors wrote the manuscript and approved the submitted version.
